# Microbial Pigments in the Food Industry—Challenges and the Way Forward

**DOI:** 10.3389/fnut.2019.00007

**Published:** 2019-03-05

**Authors:** Tanuka Sen, Colin J. Barrow, Sunil Kumar Deshmukh

**Affiliations:** ^1^TERI–Deakin Nano Biotechnology Centre, The Energy and Resources Institute, New Delhi, India; ^2^Centre for Chemistry and Biotechnology, School of Life and Environmental Sciences, Deakin University, Burwood, VIC, Australia

**Keywords:** microbial pigments, natural colorants, *Monascus* pigments, metabolic engineering, microencapsulation, food color

## Abstract

Developing new colors for the food industry is challenging, as colorants need to be compatible with a food flavors, safety, and nutritional value, and which ultimately have a minimal impact on the price of the product. In addition, food colorants should preferably be natural rather than synthetic compounds. Micro-organisms already produce industrially useful natural colorants such as carotenoids and anthocyanins. Microbial food colorants can be produced at scale at relatively low costs. This review highlights the significance of color in the food industry, why there is a need to shift to natural food colors compared to synthetic ones and how using microbial pigments as food colorants, instead of colors from other natural sources, is a preferable option. We also summarize the microbial derived food colorants currently used and discuss their classification based on their chemical structure. Finally, we discuss the challenges faced by the use and development of food grade microbial pigments and how to deal with these challenges, using advanced techniques including metabolic engineering and nanotechnology.

## Introduction

Color plays a significant role in the food production and processing sector, contributing to the sensory attribute of food. It signifies freshness, nutritional value, safety, and aesthetic value of a food, directly affecting the market value of the colored food product (1–3). Food coloring is presumed to have originated back in 1500 BCE (4). Ancient Roman and Egyptians writings show activities such as the coloring of drugs and wine. In earlier times, most of the food coloring agents were derived from natural sources such as paprika, berries, turmeric, indigo, saffron, and various flowers (5, 6). In the 1800's there was a shift toward development of synthetic colors due to their chemical stability, low production cost, and larger ranges of hue and shade. The first synthetic dye, Perkin's Mauve pigment, appeared in 1856 (4), which also lead to the discovery of other synthetic dyes. However, possible side effects of synthetic colors like hyper-activity in children, allergenicity, toxicological, and carcinogenicity problems, has led to the banning of many synthetic food colorants further leading to a transition from the use of synthetic food colors, to natural ones (7–9). An increase in the desire to label food as natural has also contributed to a decline in the use of synthetic food colorants.

Research on natural food colors has become a key area in the food industry, particularly the discovery of new natural colorants. The use of compounds as food colorants is highly regulated, whether the colors are naturally derived or synthetically produced. Organizations such as the United States Food and Drug Administration (FDA), the European Food Standards Authority (EFSA), and The World Health Organization (WHO) have advocated safe dosages for the use of these colors in food, drugs, and cosmetic items (9–11).

Food colorants exempt from certification generally include natural pigments, but no legal definition for the term ‘natural' has been adopted yet, leading to consumer, and industrial confusion. Colorants exempted from certification include a variety of pigments obtained from microbial, plant, mineral and animal sources but also include synthesized compounds that are identical to natural products, despite the common belief that colorants exempt from certification are all natural (12).

**Figure 1:**
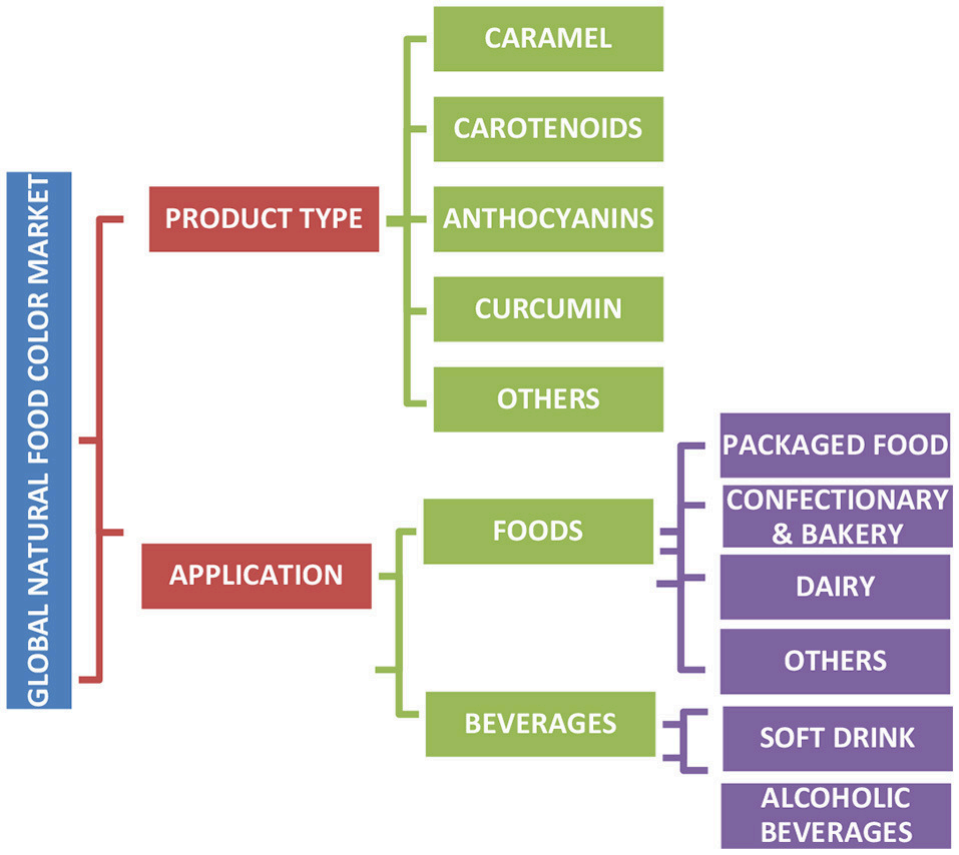
Summary of major natural pigment classes in the market and their application in foods.

Natural colors are assumed safe if they are non-allergic, non-toxic, non-carcinogenic, and biodegradable, thereby rendering no risk to the environment (5, 10). Due to the lower risk advantage of natural colors and changing perceptions of consumers to consume natural products, there is an increasing interest in the discovery of new natural colors. The consumer demand for natural colors and their growth as a category is predicted to increase by 7% annually (13–15). In recent times, natural food colors have varied applications in the food industry, with almost all major natural pigment classes being used in at least one sector of the food industry (Figure 1).

Despite the benefits, that come with using natural colors, these pigments often have drawbacks compared with synthetic colors. In many cases, potential natural pigments that can be used as food colorants present many challenges such as higher cost and lower stability.

Natural colors are primarily derived from plants, insects, mineral ores or microbial sources. Microbial colorants are preferable because of scalability ease as well as a potentially lower cost of production (4, 11). Microbial fermentation for the production of natural pigments have several benefits such as cheaper production, higher yields, easier extraction, lower-cost raw materials, no seasonal variations, and strain improvement techniques to increase natural pigment (16). These can also have health benefits like anticancer activity, antimicrobial activity and antioxidant activity (1, 17). Microbes produce a variety of pigments that can be used as food colors such as carotenoids, flavins, melanins, quinines, monascins, violacein, amongst others. They can also be used as additives, antioxidants, color intensifiers, and functional food ingredients (3, 18).

Advances in organic chemistry and metabolic engineering have enabled the mass production of microbes of interest. Studying the biosynthetic pathway for pigment production can help in understanding the roadblocks in the production of pigments and to counter that, genes can be cloned, and recombinant DNA technology can be used to increase pigment production (19, 20). Using the appropriate fermentation strategies and modifying conditions to be more suitable for the production of pigments, developing low cost processes and extraction processes, co-pigmentation strategies, have all been applied for efficient microbial pigment production. Newly emerging tools such as nanotechnology has also been effectively used in the food industry, including in pigment formulation (21). Nanotized natural food colorants derived from microbial sources can increase stability, shelf life, or solubility, leading to better delivery systems for food, and feed (22). The present review focuses on the potential of microbial pigments used as food colorants, their benefits and challenges; explores possible strategies for simplifying the process for overproduction of pigments in microbial systems, as well as the methods to improve pigment stability and formulation.

## Microbial Pigments That Can Be Used As Food Grade Colors

**Figure 2:**
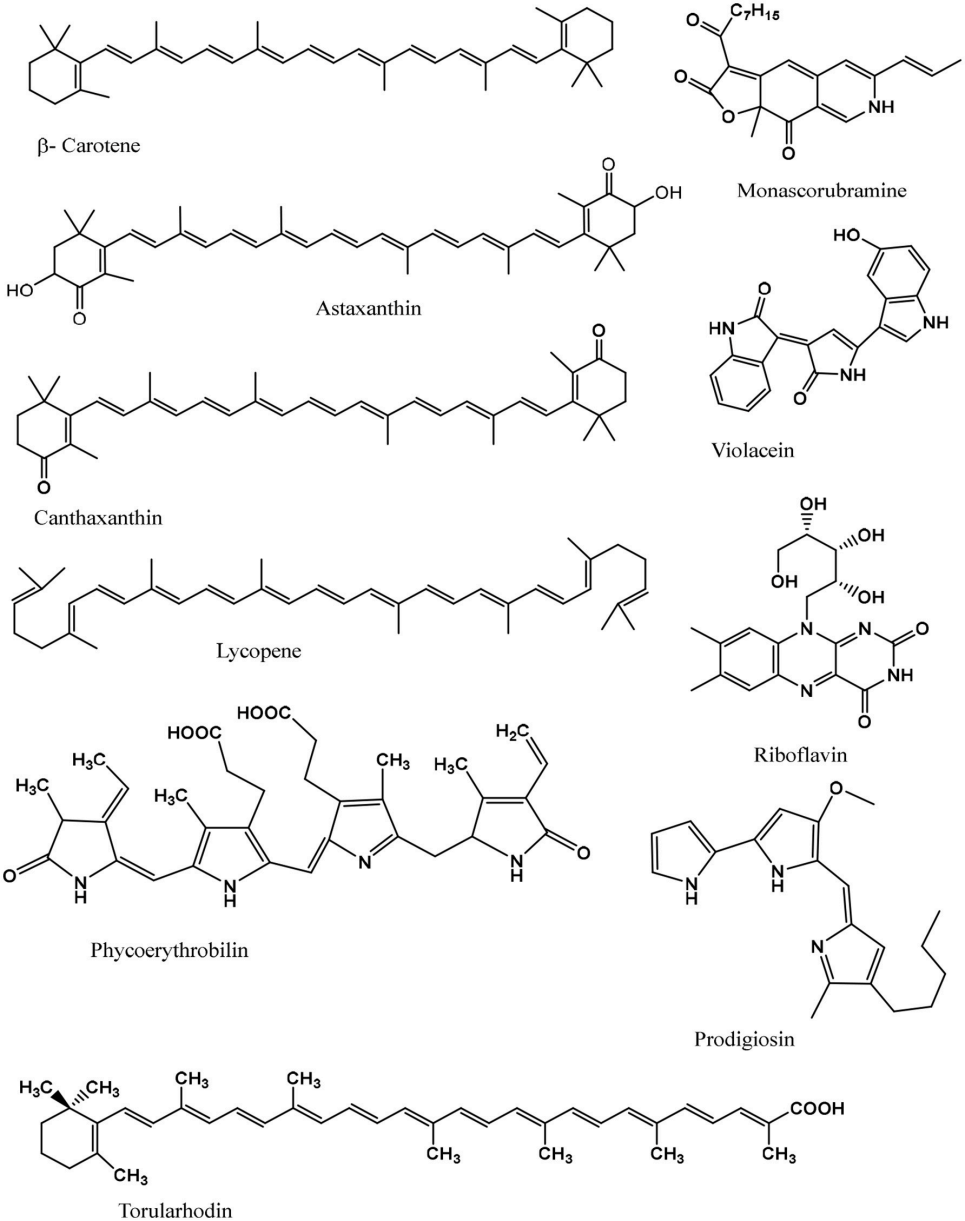
Chemical structures of some known microbial food grade pigments.

Some of the major pigments found in micro-organisms which are used as food colorants are canthaxanthin, astaxanthin, prodigiosin, phycocyanin, violacein, riboflavin, beta-carotene, melanin, and lycopene, shown in Figure 2 and a more extensive list given in Table 1. Microbial pigments can be either inorganic or organic, although organic pigments tend to be more useful as food colorants.

- **Canthaxanthin**- is an orange to deep pink colored carotenoid that is lipid soluble and a potent antioxidant. It is isolated from *Bradyrhizobium* Sepp, is a trans-carotenoid pigment, and is approved as a food colorant and used in a range of foods as well as salmon and poultry feed (100–102).
- **Astaxanthin**- is a red-orange pigment, naturally found in basidiomycetous yeast, microalgae, salmon and crustaceans, red shrimp, cray fish, feathers of some birds, and is lipid soluble (35, 37, 103–105). It's an approved coloring agent used in fish and animal foods (106).
- **Prodigiosin**- Many strains of *Serratia marcescens*, produce a red pigment, which shows antibacterial, antimalarial, antibiotic and antineoplastic activity (34, 70, 107). It has been successfully applied as coloring agents in yogurt, milk and carbonated drinks (108).
- **Phycocyanin**- is a blue pigment produced by chlorophyll A containing cyanobacteria. *Aphanizomenon flos-aquae* and *Spirulina* produces phycocyanin which is being used in the food and beverage industry as the natural coloring agent ‘Lina Blue' and is also found in sweets and ice cream (36, 109, 110).
- **Violacein**- *Chromobacterium violaceum* is one of the most prominent producers of this purple pigment, other bacterial species also produces the pigment and mostly have a purple hue. It exhibits antifungal, antibiotic, antitumor and antibacterial properties. Violacein has shown potential use in food, cosmetic and textile industries (34, 60–62).
- **Riboflavin**- Water soluble vitamin B2, is a yellow colored pigment and produced by various microorganisms. It is used in diary items, breakfast cereals, baby foods, sauces, fruit drinks, and energy drinks (34, 88–90).
- **Beta-carotene**- A red-orange colored organic pigment, mostly extracted from the beta-carotene rich algae, *Dunaliella salina* (111). Production of β-carotene through fermentation of *Blakeslea trispora* produces a pigment equivalent to pigments produced through a chemical process and is an acceptable coloring agent (24, 35, 66, 76–78). It is used in a variety of food items ranging from red to yellow in color.
- **Melanin**- Melanins are natural pigments present in animals, plants and in many micro-organisms. They are widely used in eye glasses, cosmetic, food items, sunscreen protection creams, pharmaceuticals and food items (35, 70, 96, 109, 112, 113).
- **Lycopene**- widely present and consumed in tomatoes, a brilliant red pigment consisting of carotenoid. It has been isolated from microbes like *Fusarium, Sporotrichioides, and Blakeslea trispora*, and has the potential to attenuate persistent diseases such as some types of cancers and coronary heart disease (82, 83). It is used in meat coloring in countries like the USA, Australia and New Zealand.

**Table 1:** Microbial pigments that are being used or with high potential to be used as natural food colorants.

**Sr. No**.	**Pigment**	**Color**	**Microorganism**	**Bioactivity**	Status^*^	**References**
**ALGAE AND MICROALGAE**						
1	Astaxanthin	Pink-red	*Haematococcus pluvialis* Microalgae	Antioxidant photo protectant, Anticancer, Anti-inflammatory	IP	(23–26)
2	β- carotene	Orange	*Dunaliella salina* Microalgae	Anticancer, Antioxidant suppression of cholesterol synthesis	IP	(26–29)
3	Lutein	Yellow	*Chlorella and others* Microalgae	Antioxidant	IP	(26, 30–32)
4	Phycoerythrin	Red	*Porphyridium cruentum and many other microalgae and cyanobacteria* Algae, Cyanobacteria	Antioxidant, Antitumor activity, Immunoregulatory	DS	(26, 33)
5	Phycocyanin	Blue	*Arthrospira* sp. *(formerly Spirulina* sp.*) and many other microalgae and cyanobacteria* Algae, Cyanobacteria	Antioxidant, Antitumor, Immunoregulatory	IP	(26, 34–37)
**ARCHEA**						
6	Canthaxanthin	Orange	*Haloferax alexandrines* Archea	Antioxidant, photoprotectant, Anticancer, Anti-inflammatory	NK	(38–41)
**BACTERIA**						
7	Astaxanthin	Pink-red	*Agrobacterium aurantiacum* Bacteria *Paracoccus carotinifaciens* Bacteria	Antioxidant Anticancer Anti-inflammatory, Antioxidant Anticancer	RP/IP	(23, 42)
8	Canthaxanthin	Orange	*Bradyrhizobium* spp. *Lactobacillus pluvalis*.	Antioxidant, Anticancer	RP	(38–41)
9	Granadaene	Orange-red	*Streptococcus agalactiae*	Antioxidant, detoxify ROS	DS	(43, 44)
10	Heptyl prodigiosin	Red	*α-Proteobacteria*	Antiplasmodial	DS	(45)
11	Prodigiosin	Red	*Serratia marcescens Pseudoalteromonas rubra*	Anticancer, DNA Cleavage, Immunosuppressant	IP	(46–49)
12	Phycocyanin	Blue, green	*Pseudomonas* spp.	Cytotoxicity, Neutrophil apoptosis, Ciliary dysmotility, Proinflammatory	IP	(50)
13	Rubrolone	Red	*Streptomyces echinoruber*	Antimicrobial	DS	(51–53)
14	Staphyloxanthin	Golden	*Staphylococcus aureus*	Antioxidant, detoxify ROS	NK	(54–56)
15	Tryptanthrin	Light-dark Yellow	*Cytophaga/Flexibacteria AM13,1 Strain*	Antioxidant, Anticancer	NK	(57)
16	Undecylprodigiosin	Red	*Streptomyces* sp.	Antibacterial, Antioxidative, UV-protective, Anticancer	NK	(54, 55, 58, 59)
17	Violacein	Purple	*Janthinobacterium lividum, Pseudoalteromonas tunicate, Pseudoalteromonas* spp. *Chromobacterium violaceum*	Antioxidant, detoxify ROS	NK	(60–62)
18	Zeaxanthin	Yellow	*Staphylococcus aureus, Flavobacterium* spp., *Paracoccus zeaxanthinifaciens, Sphingobacterium multivorum* Bacteria	Photoprotectant, Antioxidant	DS	(63)
**CYANOBACTERIA**						
19	Scytonemin	Reddish Brown	*Cyanobacteria* Cyanobacteria	Anti-inflammatory, Antiproliferative	NK	(64)
**FUNGI**						
20	Ankaflavin	Yellow	*Monascus* sp.	Antitumor, Anti-inflammatory	IP	(65, 66)
21	Anthraquinones	Red and other hues Known as Arpink red or Natural Red	*Penicillium oxalicum (and many other fungi)*	Antifungal, Virucidal	IP	(67–69)
22	Azaphilones	Red	*Talaromyces atroroseus Penicillium purpurogenum*	Antioxidant, Anticancer Antioxidant	DS	(70–75)
23	β- carotene	Yellow-orange	*Blakeslea trispora, Fusarium sporotrichioides, Mucor, circinelloides, Neurospora crassa, Phycomyces, Blakesleeanus*	Anticancer, Antioxidant, suppression of cholesterol synthesis	IP	(24, 35, 66, 76–78)
24	Canthaxanthin	Orange, pink	*Monascus* spp.	Antioxidant, Anticancer	NK	(34, 35, 39, 40, 79)
25	Cycloprodigiosin	Red	*Pseudoalteromonas denitrificans*	Antiplasmodial, Anticancer	DS	(80, 81)
26	Lycopene	Red	*Fusarium Sporotrichioides, Blakeslea trispora*	Antioxidant, Anticancer	RP/DS	(82, 83)
27	Monascorubramin	Red	*Monascus* spp.	Antioxidant, Anticancer	IP	(34, 84)
28	Naphtoquinone	Deep blood red	*Cordyceps unilateralis*	Anticancer, Antibacterial, Trypanocidal	RP	(70, 85–87)
29	Riboflavin	Yellow	*Ashbya gossypi*	Anticancer, Antioxidant, protection against cardiovascular diseases, in vision	IP	(34, 88–90)
30	Rubropunctatin	Orange	*Monascus* spp.	Anticancer	IP	(84, 91)
31	Xanthomonadin	Yellow	*Xanthomonas oryzae*	protection against photo damage	NK	(35, 92)
**YEAST**						
32	Astaxanthin	Pink-red	*Xanthophyllomyces dendrorhous formerly Phaffia rhodozyma*	Antioxidant, photoprotectant, Anticancer, Anti-inflammatory	DS	(93–95)
33	Melanin	Black	*Saccharomyces, Neoformans*	Antimicrobial, Antibiofilm and antioxidant	NK	(96)
34	Torularhodin	Orange-red	*Rhodotorula* spp.	Antioxidant, Antimicrobial	DS	(97–99)

* Industrial status adopted from Dufossé. (34, 42, 66).

*DS, Development stage; IP, Industrial production; RP, Research project; NK, Not Known*.

## The Benefits of using microbial pigments as food grade coloring agents

Micro-organisms are found in almost every environmental niche and have various roles in nature. They are also affiliated with food and are accountable for the fermentation of food products. Microbial pigments are a better alternative to synthetic food colors compared to plants because of their availability, non-seasonality, scalability, higher yield per hectare, and straight forward down streaming processing. Microbial pigments like that of *Monascu*s, Arpink Red (natural red- industrial name) from *Penicillium oxalicum*, β-carotene from *Blakeslea trispora* and Astaxanthin from various microbes are already used in the food industry to color foods (34, 113, 114). A lot of research has been done to lower production and processing costs for natural colors, to increase stability and shelf life, so that it can compete with the use of synthetic colors. Many of these pigments not only work as coloring agents but also impart health benefits (Bioactivity of various microbial pigments mentioned in Table 1). Micro-organisms produce an large quantities of pharmacologically and biologically active compounds that can have a diverse range of activities, including antioxidants, antimicrobial, anticancer, immuno-regulatory, and anti-inflammatory compounds.

### Antioxidant Activity

Microbial pigments like violacein, carotenoids, anthocyanins, and naphthoquinone have been shown to be potent antioxidants agents. Violacein which is a purple pigment largely produced by *Pseudoalteromonas* and *Chromobacter violaceum* (60, 62) is a powerful antioxidant which stimulates mucosal defense mechanisms to protect against oxidative damage in gastric ulcers (115, 116). *Staphylococcus aureus* produces a yellow pigment called staphyloxanthin, that prevents carbon tetrachloride induced oxidative stress in swiss albino mice (117). There are many other pigments that can act as antioxidants such as Astaxanthin, Granadaene, Canthaxanthin, Lycopene, Riboflavin, β- carotene, Torularhodin, etc.

### Anticancer Property

Anticancer activities in microbial pigments have been reported in a number of studies. These pigments can induce apoptosis, which lead to the destruction of cancerous cells. Scytonemin which is a green-yellow pigment, produced by the aquatic cyanobacteria, inhibits the action of the cell cycle regulatory protein kinase, thereby showing an antiproliferative effect (64). Prodigiosin is red pigment which is a potent anticancer compound, produced by *Serratia marcescens and Pseudomoalteromonas rubra*. It shows an apoptotic effect against human cervical carcinoma (118). Anticancer activity is shown by synthetic indole derivatives and analogs of prodigiosin *in-vitro* (119). Violacein showed cytotoxic effects on HL60 leukemia cells through a TNF signaling cascade and the activation of Caspase-8 and p38 MAPK (120). There are various pigments that can act as anticancer agents such as Astaxanthin, Canthaxanthin, Lycopene, Monascorubramin, Riboflavin, Rubropunctatin, β-carotene, Torularhodin, and others.

### Antimicrobial Activity

Many micro-organisms produce antimicrobial compounds, some of which are presently used as antibiotics. A pigment obtained from an endophytic fungus was shown to be more potent than the commercially available antibiotic Streptomycin. It was effective against bacteria like *Klebsiella pneumoniae, Staphylococcus aureus, Salmonella typhi and Vibrio cholera* (121). It is known that violacein causes growth inhibition, additionally also killing the bacteria. It also exhibits antifungal, antiprotozoal and antiviral activities (76, 77, 122). The recent emergence of antibiotic and multi drug resistant microbial strains has led to a search for new and novel compounds that can be used as antibiotics. Finding novel microbial pigments that have both pigment producing and antimicrobial properties is highly advantageous (123).

## Challenges Faced in Natural Food Colors

Even though there are many types of natural pigments from various microbial sources, the commercial development of natural pigments as food colorants is challenging. Regulatory hurdles are high for the development of any new compounds for food use, including as a colorant. The cost of using natural colors is five times more than using synthetic colors, especially when used in confectionary items, where it can be 20 times more expensive (124). Substantial quantities of raw materials are required to produce equal quantities of natural colors than synthetic colors. Higher dosages of a natural color are normally needed for the desired hue, thereby increasing the cost.

Natural pigments have many product challenges with respect to cost, application, process, and quality. Microbial pigments have a weaker tinctorial strength and may react on different food matrices, causing undesirable flavors and odors. Synthetic food colorants that the food industry came to rely on over the past 50–60 years are relatively well-behaved and consistent in their performance. Replacement of synthetic colors with natural colors in the food industry is challenging, particularly with regard to the relatively low range of natural colors approved for food use. Deodorization is another issue that arises in natural pigment products as many of the available natural pigments have an odor that is undesired in the food products. Furthermore, natural colors are generally more sensitive to light, pH, UV, temperature, oxygen, and heat, leading to color loss caused by fading and a decreased shelf life. Some natural pigments are sensitive to other ambient conditions like metal ions, proteins or organic compounds (10, 125, 126). It is well-known that vitamin C will enhance the stability of beverage products, which are colored with carotenoids like beta-carotene and paprika oleoresin, but the same vitamin will cause the degradation of anthocyanins (127).

Major microbial pigments like carotenoids, chlorophyll, anthocyanins, and others also face such limitations. Carotenoids, which are strongly colored isoprenoid plant compounds and highly conjugated, are unstable when exposed to oxygen or light (128–130). Chlorophyll undergoes rapid degradation due to enzymatic reactions or factors like light, oxygen, heat or acid, leading to the formation of chlorophyll derivatives (131). Formulation of these natural colors is challenging and methods such as micro-encapsulation can be applied to improve stability and in some cases solubility. Many fungal pigments are prohibited as natural colorants due to the presence of mycotoxins (132). It is therefore important to use non-toxic and non-pathogenic strains for natural pigment extraction. When a promising pigment-producing microbe is discovered, metabolic engineering can be used for controlled biosynthesis of the pigment and toxin production.

## Technologies for Enhancing Pigment Production

The idea is to bring microbial pigments out of petri plates and on to the market (3, 34). There is a need to find alternative colorants that are cost effective, completely natural, non-toxic and which do not produce any recalcitrant intermediates. Commercial success of a natural pigment is dependent on the investment made to obtain the final product, its regulatory approval and its influence in the market. Three key operations are important in the industrial production of natural pigments: Discovery of newer and novel alternative sources; cost effective production with uniform quality; and improved applicability (133). Rigorous trials are required to develop methods that stabilize natural pigments in different food matrices, increase shelf life, prevent the influence of various environmental parameters on the pigment, finding inexpensive organic substrates for the growth of the pigment producing micro-organisms and making the fermentation process more cost effective (134).

### Newly Developed Smarter Screening Methods

There are many new advances in the quick and easy detection of microbial pigments. One of the best examples is the condensed handheld Raman spectrometer, used for detecting pigments with the help of a 532 nm excitation laser. This can detect common and uncommon carotenoids, bacterioruberin and other known pigment compounds. This hand-held device has been used to identify microbial pigments in various ecological niches including halophilic micro-organisms (135, 136).

Intelligent screening also includes having prior knowledge of the toxic metabolite pathway of the pigment producer, so that toxic and pathogenic pigment producers can be ruled out or manipulated for food coloring purposes. *Fusarium venenatum* produces a mycelial food product QuornTM, which is also known to produce a cytotoxic compound called 4, 15- diacetoxyscirpenol (137).

Mass spectrometry with electrospray ionization can also be used for faster identification of pigment producing fungal strains and for grouping them in classes and subclasses (138). More than 15,000 microbial metabolites are already known and so rapid dereplication and identification of known compounds is important. HPLC, mass spectrometry, LCMS, nuclear magnetic resonance (NMR), and UV-VIS spectra can be applied to the rapid identification of known compounds even within relatively complex mixtures, without the need for individual compounds purification (139).

### Strain Development and Fermentation

There are several challenges linked to scaling up the production of microbial pigments, but recent advances in technology has helped in somewhat overcoming these challenges. The use of fermentation tanks for large scale production of pigments, the use of strain improving techniques and strain development through random mutagenesis and multiple selection rounds has helped to develop a cost effective and industrially viable production process for pigments and other natural compounds. Strain development is important because the pigments produced by wild type strains are often too low in quantity and take longer fermentation times, making the process uneconomical. Strain improvement is done by common mutagens like 1-methyl-3-nitro-1-nitrosoguanidine (NTG), Ethyl methyl sulfonate (EMS) and Ultraviolet (UV), which can lead to a several-fold increase in pigment production (140–142).

Medium optimization is an important process for maximizing yield of the fermentative product. Optimizing the medium includes controlling operating conditions like temperature, pH, aeration, agitation, and media components. Response surface methodology (RSM) is an effective approach for the process optimization of pigment production. This solves the multivariate data obtained to solve multivariate equations, thereby reducing the number of experimental trials needed to evaluate multiple variables (142, 143). Su et al. developed an optimal medium composition, which can be used for culturing *Serratia marcescens* in the production of prodigiosin. Sucrose and glycine were added as a carbohydrate and energy source, which increased the production of prodigiosin by 2.12–2.15 folds. Inorganic supplementation with KH2PO4 accelerated cell growth, leading to the increased production of prodigiosin (144). To develop an economical production process, efficient fermentation design and standardization of the medium is important. Application of statistical techniques can result in an improved output response and can reduce variability and overall costs (21).

### Cost-Effective Downstreaming

Developing more cost-effective recovery and separation techniques for microbial pigments are also needed. Large-scale separation and recovery of pigments using conventional methods is costly. Extraction using organic solvents is a complicated and time-consuming process, in which substantial amounts of organic solvents are exhausted while the yield of the high purity product can be extremely low. In addition, using solvents other than water and ethanol can defeat the purpose of obtaining a natural pigment for regulatory purposes, since most organic solvents are not natural. The technique of using non-ionic adsorption resins for an efficient separation and purification has been applied to many nucleic acids, organic acids, peptides, and others (145, 146). These resins have a high loading ability, thereby helping in recovering of compounds in large quantities. In addition, these resins can directly be used to adsorb compounds from the culture broth. It helps in lowering the cost of separation, by lessening the consumption of extraction solvents and increasing its reusability. An efficient method for prodigiosin separation and purification was described by Wang et al. who used non-ionic resins directly from the culture broth, thereby eliminating the cell separation step, yielding a concentrated and semi-purified product (147).

### Metabolic Enginerring

Recent developments in molecular biology and metabolic engineering have led to the cloning of genes responsible for pigment biosynthesis and enabled overproduction of these pigments by gene manipulations. Pigment biosynthetic pathways have been extensively studied and engineered to overproduce a pigment and to change the pigments' molecular structure and color. Blue pigment Actinorhodin, produced by *Streptomyces coelicolor*, has been genetically manipulated to produce a related bright yellow polyketide known as kalafungin, that is used to produce an antraquinone, which is a reddish-yellow color (148, 149). Heterologous expression has been used to develop cell factories to efficiently produce pigments by expressing biosynthetic pathways from novel or known pigment producers (150, 151).

Understanding the biosynthetic pathways for microbial pigments is an extremely important starting point, followed by identifying genes and the gene cascades responsible for pigment production, then engineering these genes for over production. Cloning the genes responsible for pigment biosynthesis into microbial vectors, like bacterial or yeast cells, has become a cost-effective and more economical industrial production process. Industrially reliable micro-organisms such as *E.coli, Bacillus subtilis, Pseudomonas putida, Corynebacterium glutamicum, and Pichia pastoris*, can be used to developof tailor-made recombinants, genetically engineering the production of pigments (152).

Techniques like selected and random mutagenesis are used to obtain hyper-producing strains, and for this, chemicals and physical methods such as 1-methyl-3-nitroguanidine, antymicin A, or Ethyl methane sulfonate and Gamma radiation and UV light are employed (153). Carotenogenic genes from *Xanthophyllomyces dendrorhous or Erwinia uredovora or Agrobacterium aurantiacum*, yeasts like *Candida utilis* and *Saccharomyces cerevisiae* are genetically manipulated to produce carotenoids like lycopene or β-carotene or astaxanthin (154–156). At this time, engineering genes responsible for carotenoid pigments, has been limited to non-carotenogenic micro-organisms like *C.utilis* or *S. cerevisiae*. There is almost no published data on the metabolic engineering of wild type carotenoid producers like *Dunaliella salina, B. trispora* and *R. mucilaginosa*. Wang et al. (157) used metabolic engineering and mutagenesis to enhance carotenoid production in *R.mucilaginosa* KC8, which produces carotenoids, mainly β-carotene and torularhodin (157). Grewal et al. (158) described betaine production in a heterologous microbial host Saccharomyces cerevisiae, using glucose as a substrate. They also established that novel betalain derivatives could be obtained by feeding different amines in the culture (158).

In the case of *Monascus*, three polyketide pigments are produced namely Citrinin, red pigments and monacolin K (159, 160). Various techniques have been tried to decrease the production of citrinin, a mycotoxin, and to increase the production of the red pigment. Changes in the nitrogen composition, dissolved oxygen, pH, and genetic alterations are some of the various techniques tried to minimize citrinin. The polyketide synthase gene responsible for biosynthesis of citrinin has been studied in *Monascus purpureus*. In the industrial strain *M. purpureus* SM001, the polyketide synthase gene pksCT has been successfully cloned to eliminate citrinin production (161–163).

## Metabolic Engineering Using the CRISPR-Cas9 System

CRISPER-Cas9 has created a trend and various laboratories are using the technology for newer applications in biology, especially genome engineering. CRISPR stands for Clustered Regularly Interspaced Short Palindromic Repeats. It consists of two key components that brings about the change in DNA, the first being the enzyme Cas9, which acts like molecular scissors and makes double stranded cuts at the target location, helping in adding, or removing pieces of DNA. The second component is a piece of RNA, also known as the guide RNA, which is a pre-designed sequence of about 20 bases, and which is located inside a longer RNA scaffold. This scaffold binds to the target DNA sequences and the guide RNAs directs the Cas9 enzyme to make cuts at the right point in the genome. Due to the cuts being made, the cell activates its DNA repair machinery and tries to repair the damages, which can be efficiently used for introducing changes to one or more genes in the genome (164). Hence CRISPER-Cas 9 system can be very well used for metabolic engineering in bacteria, yeast and fungi to make them cellular factories for cost efficient production of natural food colors (165).

CRISPER- Cas9 has been efficiently used in the production of industrially important metabolite compounds. It can be used in a wide variety of bacterial cells such as *Corynebacterium, Escherichia coli, Pseudomonas, Staphylococcus, Bacillus, Clostridium, Lactobacillus, Mycobacterium and Streptomyces*, genetically modifying them to produce metabolites such as biofuel, biochemical, pharmaceutical precursors, or any other significant metabolite (166). The CRISPER system has been used in the industrial yeast *Saccharomyces cerevisiae*, as it is one of the most noticeable cell factories for the industrial production of a large number of products. It can also be engineered to produce natural colors, if a color-producing gene is inserted into its genome using the CRISPER-Cas9 system (167).

Metabolic engineering in filamentous fungi have been extremely tough due to various reasons such as a lack of genetic markers and even when they are available, it remains a tedious process because of low gene-targeting frequencies. The CRISPER-Cas9 system has been employed in *Neurospora crassa* (168), *Aspergillus nidulans* (169) and in several other species of filamentous fungi such as *Magnaporthe oryzae* (170) and *Trichoderma reesei* (171). Nielson et al. have developed a system for *Aspergillus nidulans*, harboring the CRISPER-Cas 9 system that can potentially be applied in many fungal systems with close to no adaptation. They showed that is was useful in an extensive array of filamentous fungi (151). They even used the same system in *Talaromyces atroroseus*, which is a major natural red color producer in the food industry. Recently, the CRISPER-Cas9 system was used in *Penicillium chrysogenum* (172) showing a rapid improvement of engineering filamentous fungi. Limited research studies on using CRISPER-Cas9 in micro-organisms for pigment production exist. More research is required to optimize the use of the CRISPER system for this application.

## Addressing Instability of Natural Pigments

To be industrially useful, microbial pigments need to be stable against environmental factors like light, pH, temperature, UV, and food matrices. Many microbial pigments are rendered useless because of their instability against ambient conditions and have short shelf life. There are various techniques available that can produce a more stable natural pigment, which has a higher shelf life and market value in terms of the cost-effective stability measures taken.

### Microencapsulation, Nanoemulsions, and the Formation of Nanoformulations

Micro-encapsulation and nano-formulations can be applied to stabilize, improve solubility and deliver natural pigments to food matrices. Natural colors like anthocyanins and carotenoids, have stability issues in various environmental conditions and also present solubility problems in some matrices (173). Micro-encapsulation can be defined as packing any solid, gas or liquid in sealed capsules of sizes ranging from millimeters to nanometers (174). The core or the active compound becomes the packaging material, in this case the microbial pigment and the packaging material, is called the wall or shell material (174). The wall material used should have emulsifying properties, low viscosity, be biodegradable, should have film forming properties, should resist GIT, be low cost and should show low hygroscopicity (175). There are various wall materials that are currently used to encapsulate microbial pigments for use as food color such as maltodextrins, modified starch, inulin, furcellaran and others (176).

Encapsulated colors are easier to handle, have better solubility, and show improved stability to ambient conditions, leading to an increased shelf life. The wall material protects the active core material from light, temperature, oxygen, humidity, and matrix interactions. The major objectives of encapsulating microbial pigments and their application in the food industry are: Increasing shelf life, protecting the core material from undesirable environmental conditions, ease, and flexibility of handling and controlling the release time of the pigment and suppressing any type of aroma or flavor. Various methods of micro-encapsulation are available. Prominent examples used in the food industry are spray-drying, coacervation, freeze-drying and emulsion formation. There are numerous reports on encapsulated microbial pigments, such as anthocyanin, in which maltodextrin has been micro-encapsulated as the wall material, using spray-drying (177). B-Carotene has been reported to be encapsulated in modified starch as the wall material using freeze drying (178). These encapsulated colors have also been applied in food and beverage systems like yogurt, soft drinks, cake, and others, and these have shown to be stable and effective (179–181).

Nano-encapsulation or nano-emulsions are droplet size, 100 nm or less, and can also be prepared to encapsulate microbial pigments. Nano-emulsions contain three constituents, water, oil, and emulsifier. The addition of an emulsifier is the most critical step in forming a nano-emulsion, as it helps to decrease the tension between the water and oil phases of the emulsion. It also stabilizes the nano-emulsion by negating the steric hindrance and repulsive electrostatic interactions. The emulsifiers used are mostly surfactants, but proteins and lipids are also used. Compared to micro- and macro-emulsions, nano-emulsions have improved applications because of their large surface area per unit, stronger kinetic stability and resistance to any chemical or physical change (182). Importantly, nano-emulsions, and nano-capsules are small enough to be invisible in solutions and are therefore useful vehicles for the dispersion of poorly water-soluble pigments in aqueous solutions. Creating nano-emulsions for food colorants can provide various advantages. The small sized droplets that are made in the formation of nano-emulsions provide a much greater surface area and therefore greater absorption. These nano-emulsions also are non-irritant in nature and non-toxic, making them suitable for food industry use. These can also be formulated in a wide range of formulations such as creams, liquids, sprays, and foams. Nano-emulsions create no undesired taste to the food particle and stabilize the colorant within the emulsion from all environmental conditions (183). Nano-emulsions of food colorants can significantly decrease the amount of colorant needed to obtain the desired color food particle, thereby proving to be cost effective. Various studies of nano-emulsion formation of β-carotene have been carried out; Yuan et al. studied the size and stability of nano-emulsions with b-carotene against temperature, pH and surfactant type. Qian et.al. prepared nano-emulsions with b-carotene and stabilized them with beta-lactoglobulin, a biocompatible emulsifier (184).

## Conclusions and Future Perspectives

Natural foods are an important and growing food category that require natural ingredients and additives. Subsequently, there is a great demand to replace synthetic pigments with natural pigments in food and beverages. Microbial sources are particularly useful as they can be scaled-up and are more readily manipulated than plants or insects. Development and integration of advancements like strain development in fermentations, systems biology, metabolic, and protein engineering, can make a substantial difference in both the quality and quantity of natural food colors. Efficient fermentations include predictable yields and no external influence of the climate or environment. However, further research is required to optimize pigment characteristics, like composition and yield, by finding the most optimized parameters for growth, use of genetically modified organisms to enhance production, and also the presence of various elicitors for pigment production (185).

Metabolic engineering is useful but has its own regulatory challenges. In terms of technology, metabolic engineering can improve product yields, enable the transfer of pathways from slow growing organisms to faster growing ones, and enable directed biosynthesis of analogs of a pigment, to modify color or other properties. Cell factories can be created by utilizing CRISPER-Cas9 and heterologous expression of biosynthetic pathways from known or novel pigment producers can provide useful strategies (150, 151). Poor stability or low solubility of natural food colorants can be addressed by techniques like micro-encapsulations and nano-formulations, enabling a wider application of microbial pigments to various food matrices. Encapsulated colors are easier to handle, have better solubility and show improved stability to ambient conditions, which lead to an increased shelf life. Nano-emulsions can be used to improve solubility and provide invisible particles that are useful in the coloring of clear and semi-clear beverages.

The current range of natural colors that can be added to foods is relatively small compared to the large range of synthetic colors. However, demand for natural foods and natural colors is increasing. The discovery of new and novel natural colors is therefore important, as is the development for technologies to improve the cost effectiveness of production and formulation of natural pigments. New natural sources to obtain pigment producing micro-organisms are required, as well as process improvements to make these strains more cost competitive with synthetic pigments. The technology required includes the development of low-cost organic substrates for the growth of pigment producing microbes, newer methods to increase the production of pigments, and stabilizing methods for improving pigment application. Research on natural pigments should focus on obtaining a wider variety of hues, using pigments with health benefits, increasing pigment shelf life, and lowering production costs.

## Author Contributions

TS drafted and edited the manuscript. CB critically revised the manuscript. SD provided critical revisions and approved the final version of the manuscript for publication.

### Conflict of Interest Statement

The authors declare that the research was conducted in the absence of any commercial or financial relationships that could be construed as a potential conflict of interest.
